# Comparative metagenomic analyses reveal viral-induced shifts of host metabolism towards nucleotide biosynthesis

**DOI:** 10.1186/2049-2618-2-9

**Published:** 2014-03-26

**Authors:** Hagay Enav, Yael Mandel-Gutfreund, Oded Béjà

**Affiliations:** 1 Faculty of Biology, Technion Israel Institute of Technology, Haifa 32000, Israel

**Keywords:** Metabolic networks, Metabolism, Nucleotide biosynthesis, Phage, Virus

## Abstract

**Background:**

Viral genomes often contain metabolic genes that were acquired from host genomes (auxiliary genes). It is assumed that these genes are fixed in viral genomes as a result of a selective force, favoring viruses that acquire specific metabolic functions. While many individual auxiliary genes were observed in viral genomes and metagenomes, there is great importance in investigating the abundance of auxiliary genes and metabolic functions in the marine environment towards a better understanding of their role in promoting viral reproduction.

**Results:**

In this study, we searched for enriched viral auxiliary genes and mapped them to metabolic pathways. To initially identify enriched auxiliary genes, we analyzed metagenomic microbial reads from the Global Ocean Survey (GOS) dataset that were characterized as viral, as well as marine virome and microbiome datasets from the Line Islands. Viral-enriched genes were mapped to a “global metabolism network” that comprises all KEGG metabolic pathways. Our analysis of the viral-enriched pathways revealed that purine and pyrimidine metabolism pathways are among the most enriched pathways. Moreover, many other viral-enriched metabolic pathways were found to be closely associated with the purine and pyrimidine metabolism pathways. Furthermore, we observed that sequential reactions are promoted in pathways having a high proportion of enriched genes. In addition, these enriched genes were found to be of modular nature, participating in several pathways.

**Conclusions:**

Our naïve metagenomic analyses strongly support the well-established notion that viral auxiliary genes promote viral replication via both degradation of host DNA and RNA as well as a shift of the host metabolism towards nucleotide biosynthesis, clearly indicating that comparative metagenomics can be used to understand different environments and systems without prior knowledge of pathways involved.

## Background

Viruses are the most abundant biological entities in the marine environment [1-3], with observations of millions of viral particles per single drop of water [4]. The high abundance of viruses combined with evidence that viral particles are a major source of planktonic mortality [5-7] and horizontal gene transfer [8] demonstrate the immense influence of the virosphere on the ecology and biology of the marine environment.

One of the key aspects of viral evolution is the acquisition of host genes and their fixation in the viral genomes. It was suggested that such genes improve viral fitness [9,10] by supporting key steps in host metabolism and were therefore termed ‘auxiliary metabolic genes’ [11]. For example, the contribution of the viral *psbA* gene, which is a key factor in host photosynthesis, was shown to increase the viral genome number produced during the lytic cycle of cyanophage P-SSP7 [12]. The identification of auxiliary metabolic genes found in viral genomes is enabling a deeper understanding of the functions that increase viral fitness and the mechanism in which these functions are promoted. Recent studies have provided evidence of viral acquisition of host genes encoding enzymes in key metabolic pathways, such as phosphate metabolism [13-15], nitrogen metabolism [16], photosynthesis and pigment biosynthesis [9,10,13,17-22], pentose phosphate pathway [23-25], nucleic acid synthesis [26] and others [25]. It was suggested that these genes play a role in overcoming biochemical bottlenecks in metabolic processes [11], thus increasing viral fitness by enhancing specific metabolic processes during infection. Notably, while increasing viral fitness, newly acquired genes are fixed in the viral genomes at the expense of larger genome sizes, and thus only the most essential genes are expected to be enriched in the viral genomes.

In this work, two viral metagenomic datasets originating from the Global Ocean Survey (GOS) project [27] and the Line Islands metagenomic biomes project [28] were analyzed to detect statistically-enriched host-like metabolic genes in viral reads. To investigate the potential contribution of the enriched genes and pathways found to viral fitness, we mapped the enriched ortholog groups of genes onto a “global metabolism network.” The global metabolism network was constructed by connecting metabolic pathways sharing the same metabolic compounds. Our analysis demonstrates that the viral-enriched pathways have higher connectivity values and are closely associated with the purine and pyrimidine metabolism pathways, which are among the most enriched pathways in the viral metagenome. Furthermore, we show that the enriched ortholog groups are often clustered in specific parts of individual metabolic maps and enhance consecutive reactions. Overall, our data strongly support the hypothesis that the closely associated metabolic pathways play a role in shifting the host metabolism towards purine and pyrimidine biosynthesis to increase viral fitness.

## Methods

### Datasets

Two marine viral metagenomic datasets were used in this study. The first dataset is based on data generated in the VirMic project [25] (http://www.cs.technion.ac.il/~itaish/VirMic/) and includes a collection of GOS scaffolds that are considered to be of viral origin (and contain microbial genes). Briefly, the metagenomic scaffolds included in the VirMic dataset were identified using a reciprocal blast approach against the Refseq viral and microbial databases [29]. In the final screening phase of the VirMic pipeline, only scaffolds containing either three genes, out of which the two edge genes are considered to be of viral origin, or at least four genes, out of which three or more are considered to be of viral origin, were included. The original viral metagenome was comprised of metagenomic scaffolds that were converted to the corresponding reads using the “VirMic.scaffolds.gff” file and further filtered to remove possible contaminations.

The second set of data originates from the Line Islands metagenomic biomes project [28]. These metagenomes were chosen as they allow a comparative analysis of the Line Islands viromes and microbiomes. This comparison is possible as each geographical site was sampled twice using different filtering approaches, resulting in viral and microbial datasets using the same sequencing method. Line Islands viromes analyzed were: Line Is Kingman, Line Is Christmas, Line Is Palmyra, and Line Is Tabuaeran (SEED accession: 4440036.3, 4440038.3, 4440040.3, and 4440280.3). Line Islands microbiomes analyzed were: Line Is Kingman, Line Is Christmas, Line Is Palmyra, and Line Is Tabuaeran (SEED accession: 4440037.3, 4440039.3, 4440041.3, and 4440279.3).

### Advantages and limitations of GOS-based datasets and viral-size fraction metagenomes

The major analysis is based on the classification of GOS metagenomic scaffolds, as was performed by Sharon et al. [25]. Analysis of GOS scaffolds presents several problems characteristic to this type of data. The main drawback of the GOS scaffolds results from the assembly procedure that was chosen to map the DNA reads; reads from different GOS stations (i.e., different geographical locations) were often assembled onto a single scaffold. While this tactic results in longer scaffolds and higher assembly rates, it might induce biases resulting from misassembled scaffolds. Another disadvantage of the dataset is the limited knowledge of viral diversity in oceans. The VirMic dataset was generated in 2011 and is therefore based on annotations updated in the same year. Recent discoveries, such as the identification of viruses infecting SAR11 and SAR116 bacteria [30,31] and the following updates to existing databases, might alter the results of the classification performed by Sharon et al. [25]. The pipeline described in this study could be used reliably to detect dominating trends in the viral metagenome such as pathways with high enrichment scores. Nevertheless, with regard to specific genes, a conservative approach should be taken, including a manual examination of the relevant GOS scaffolds and reads, in order to verify, once again, the viral origin of the specific gene. An important advantage of GOS-based data is the length of DNA reads, ~900–1000 bp/read. The length of these reads allows accurate functional annotation of the dataset.

Datasets derived from viral-size fraction metagenomes, such as the Line Islands dataset used in this project, introduce some biases and difficulties. The most prominent problem is the contamination with bacterial DNA [32], presumably derived from Gene Transfer Agents [8] or bacterial vesicles [33]. Moreover, the short length of the reads in those datasets (~100 bp) often results in poor functional annotations and a high rate of false positive results.

### Annotation, enrichment calculation, and pathway mapping

Updated annotated files (August 27, 2012) of the entire GOS reads against the KEGG database were downloaded from the MG-RAST website [34] (http://metagenomics.anl.gov/). In the files downloaded from MG-RAST, some reads were clustered and appeared in the following format: aa90_xxx. We converted these clusters to their corresponding reads to be employed in the downstream analysis using the 550.cluster.aa90.mapping files for each GOS station. Only annotations with e-values <1e-5 were used for downstream analysis. Annotation files for the Line Islands viromes were also downloaded from the MG-RAST website. For the Line Islands metagenomes, we considered only annotations with e-values <1e-5 and sequence identity >75%, as the shorter read length of these datasets might result in miss-annotations.

We counted the appearances of KEGG Orthologs (KO) in the reads associated with VirMic in the entire GOS dataset. Functional enrichment was determined using the hypergeometric distribution probability test (Equation (1)):

(1) $$p\left(X=k\right)=\frac{\left(\begin{array}{c} K\\ k \end{array}\right)\;\left(\begin{array}{c} N-K\\ n-k \end{array}\right)}{\begin{array}{c} N\\ n \end{array}}$$

where N is the number of KO annotations found in the GOS project, n is the number of KO annotations in the VirMic dataset, K is the number of reads annotated as a specific KO in the GOS project, and k is the number of the reads annotated as the same specific KO in the VirMic dataset. KOs with a *P* <1 × 10^-4^ were considered enriched (Additional file 1: Table S1); these KOs were mapped to KEGG metabolic maps using publically available data for each KO in the KEGG website. In order to analyze the Line Islands virome, we modified our pipeline using the Fisher exact test instead of the hyper geometric test, as the Line Islands metagenomes are divided into two corresponding sets (microbiome and virome) and not into set and subset, as in the case of the GOS and VirMic.

The pathway enrichment score was developed for this study and is calculated for pathways that contain viral-enriched KOs. The enrichment score is defined in Equation (2):

(2) $$\mathrm{ES}=\frac{N*\sum_{N}^{1}\left(‒\log_{10}\left(\mathrm{Pn}\right)\right)}{T}$$

where ES is the pathway enrichment score, N is the number of KOs that participated in the pathway and were found enriched in the viral metagenome, Pn is the hypergeometric *P* value for each enriched KO, and T is the total number of KOs in the specific KEGG pathway.

### Global metabolism network generation and analysis

To generate the global metabolism network, we used the 148 KEGG metabolic pathways that comprise the “metabolic pathways” in the KEGG database (KO01100). We treated each metabolic pathway as a node in the network; if one of the products of pathway A is compound c1 and the substrate of pathway B is compound c1, we created an edge connecting the two nodes, employing a similar approach to the one performed by Kreimer et al. [35]. The edges in this network were defined as having equal weights and are non-directional. Network visualization and analysis was performed using the cytoscape software platform [36,37].

### Metabolic motif identification and examination

Metabolic motifs were defined as chains of at least four metabolic reactions promoted by ≥3 viral-enriched KOs. The number of metabolic reactions promoted by non-enriched KOs was limited to one per motif. To test for the statistical significance of metabolic motifs within the specific pathways, we multiplied the *P* value for each reaction in the motif according to the binomial distribution (Equation (3)):

(3) $$P_{y}\left(y\right)=\left(\begin{array}{c} n\\ y \end{array}\right)\;P^{y}{\left(1-P\right)}^{n-y};y=0,1\ldots ,n$$

where P is the probability of finding a reaction promoted by a viral-enriched KO in a specific pathway, *n* is the total number of reactions containing compound c (excluding the previous reaction of the metabolic motif), and *y* is the number of reactions promoted by enriched KOs that contain compound c.

## Results and discussion

### Detection of viral-enriched host-like metabolic genes

**Figure 1:**
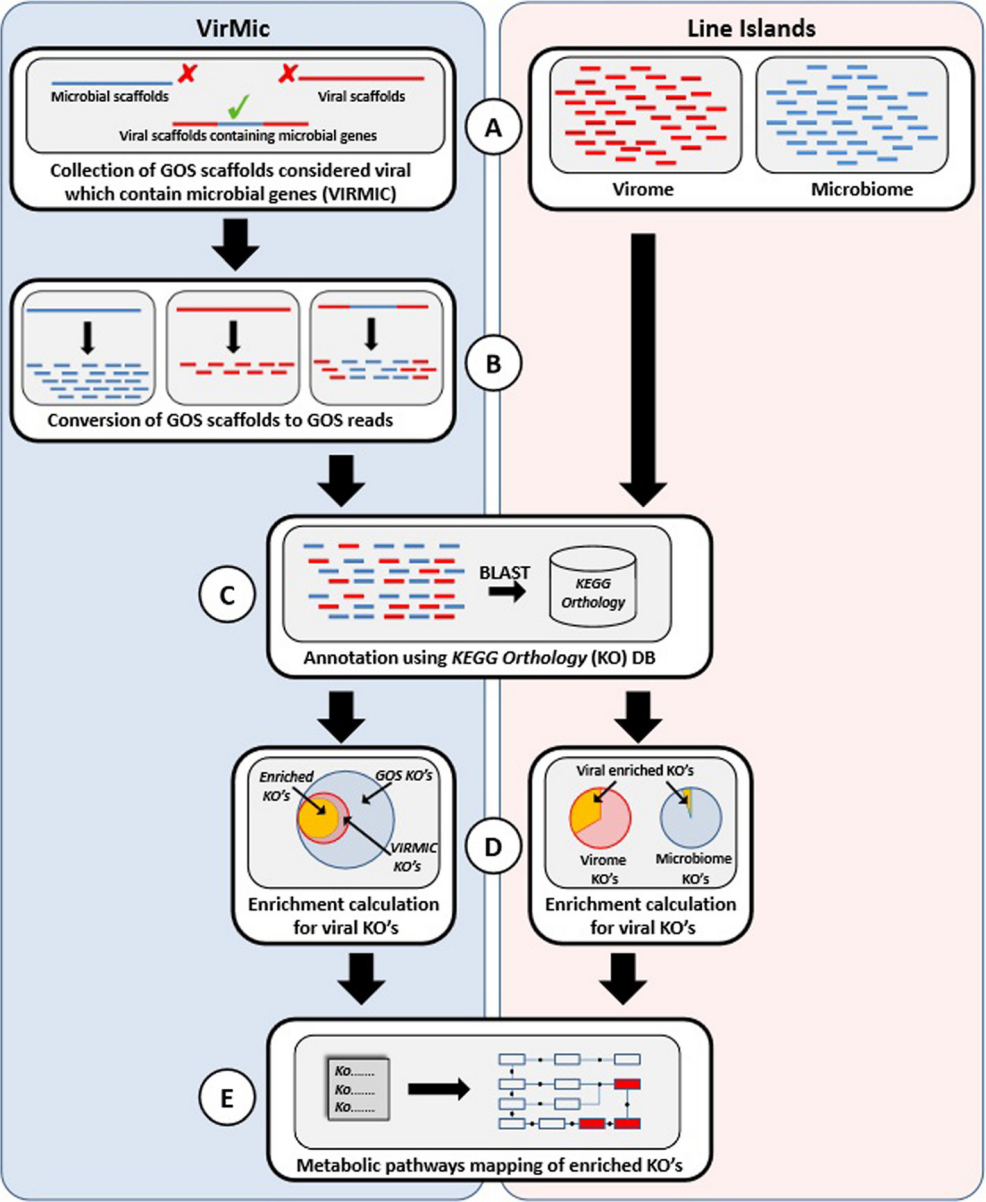
**The pipeline for the identification of enriched metabolic ortholog groups and pathways within marine viral metagenomes. (A)** VirMic dataset (left) is a collection of metagenomic scaffolds of the GOS project that are considered to be of viral origin and contain microbial metabolic genes. Line Islands metagenomes (right) are composed of four microbial-size fraction metagenomes and the corresponding four viral-size fraction metagenomes. **(B)** VirMic scaffolds were converted to the original reads to allow better estimation of gene abundance. **(C)** All metagenomic reads were annotated based on the KEGG Orthology database. **(D)** Hypergeometric enrichment analysis was performed to identify enriched KOs in the VirMic subset (left). Fisher exact test was applied in order to detect KOs enrichment in the Line Islands virome, compared to the corresponding microbiome (right). **(E)** Enriched KOs were mapped to KEGG metabolic pathways to detect enriched viral pathways.

In order to define the enriched set of metabolic genes carried in viral genomes, we used the previously published Line Islands metagenomes, including four viral-size fraction and four microbial-size fraction metagenomes, each pair sampled at the same geographical locations [28]. We also used the VirMic dataset [25], which is a collection of GOS scaffolds [27] defined as being of viral origin that also contain microbial genes. In order to assess the abundance of genes in the environment, we disassembled all GOS and VirMic scaffolds to their corresponding reads (Figure 1). Furthermore, the entire GOS reads dataset was annotated against the KEGG Orthology database [38,39] using the MG-RAST server [34], resulting in ~2,680,000 annotations out of ~9,700,000 DNA reads. The same procedure was performed on the Line Islands metagenomes, resulting in ~16,000 microbiome annotations and ~33,000 virome annotations. The KEGG Orthology database consists of manually curated ortholog groups (KEGG Orthologs, KO) that correspond to KEGG pathway nodes. Hypergeometric enrichment analysis was performed for each KO in each of the viromes studied to detect ortholog groups that are over-represented in the viral metagenome compared to the entire GOS metagenome (when analyzing VirMic) or to the Line Islands microbiome (when analyzing the Line Islands virome). Enriched KOs were mapped to KEGG metabolic maps to better understand the role of the viral-enriched enzymes in specific metabolic pathways. Overall, 68 enriched KOs were found in VirMic and were mapped to 53 KEGG pathways (Table 1). In the Line Islands virome, 131 KOs were found enriched and were mapped to 98 KEGG pathways.

**Table 1:** Metabolic pathways found to be enriched in VirMic

**Pathway**	**Name**	**Enrichment score**	**KO’s enriched**	**KO’s in the pathway**	**KO’s**
*ko00240*	Pyrimidine metabolism	4.058130678	10	165	K00525, K00526, K00762, K01493, K02323, K02335, K03006, K03465, K10807, K10808
*ko03030*	DNA replication	2.967323268	4	51	K02314, K02335, K03111, K10755
*ko00230*	Purine metabolism	2.475427099	10	251	K00525, K00526, K00860, K00939, K01768, K02323, K02335, K03006, K10807, K10808
*ko00051*	Fructose and mannose metabolism	2.337880458	5	77	K00971, K01623, K01711, K01809, K02377
*ko00670*	One carbon pool by folate	1.568059864	2	27	K00605, K03465
*ko03430*	Mismatch repair	1.346727877	2	45	K03111, K10755
*ko00540*	Lipopolysaccharide biosynthesis	1.215485245	2	34	K00979, K03111
*ko00480*	Glutathione metabolism	0.930033621	2	39	K10807, K10808
*ko00710*	Carbon fixation in photosynthetic organisms	0.866466913	2	36	K01623, K01808
*ko00520*	Amino sugar and nucleotide sugar metabolism	0.740996687	4	129	K00971, K01711, K01809, K02377
*ko03420*	Nucleotide excision repair	0.699379877	2	48	K02335, K10755
*ko00680*	Methane metabolism	0.679862658	4	224	K00124, K01079, K01623, K08350
*ko03440*	Homologous recombination	0.626567452	2	59	K02335, K03111
*ko00030*	Pentose phosphate pathway	0.547242261	2	57	K01623, K01808
*ko00061*	Fatty acid biosynthesis	0.533333333	1	30	K02371
ko00195	Photosynthesis	0.528989459	2	63	K02703, K02706
*ko00770*	Pantothenate and CoA biosynthesis	0.5	1	32	K00606
*ko00630*	Glyoxylate and dicarboxylate metabolism	0.425930951	2	76	K00124, K08350
ko04113	Meiosis - yeast	0.388260624	2	99	K01768, K02604
*ko00260*	Glycine, serine and threonine metabolism	0.351753529	2	85	K00605, K01079
*ko03060*	Protein export	0.256223722	1	39	K03217
*ko00521*	Streptomycin biosynthesis	0.175276585	1	18	K01710
*ko04122*	Sulfur relay system	0.163056569	1	21	K11179
*ko00910*	Nitrogen metabolism	0.162848544	2	117	K00459, K00605
*ko04112*	Cell cycle – Caulobacter	0.162846923	1	31	K02314
*ko00071*	Fatty acid metabolism	0.162080418	1	49	K06445
*ko03070*	Bacterial secretion system	0.135036827	1	74	K03217
*ko00010*	Glycolysis/Gluconeogenesis	0.133035339	1	91	K01623
*ko00190*	Oxidative phosphorylation	0.127347781	2	212	K05575, K05580
ko03013	RNA transport	0.119402985	1	134	K03257
*ko02020*	Two-component system	0.11066639	2	377	K02040, K08350
ko01055	Biosynthesis of vancomycin group antibiotics	0.108792363	1	29	K01710
ko00523	Polyketide sugar unit biosynthesis	0.098593079	1	32	K01710
*ko00330*	Arginine and proline metabolism	0.091966665	2	131	K00472, K01572
*ko00920*	Sulfur metabolism	0.085907839	1	34	K00860
*ko00270*	Cysteine and methionine metabolism	0.075367382	1	70	K00558
*ko03410*	Base excision repair	0.06057902	1	41	K02335
ko04110	Cell cycle	0.060121472	1	106	K02604
*ko05152*	Tuberculosis	0.054554001	1	131	K02040
ko04111	Cell cycle – yeast	0.054007424	1	118	K02604
ko05111	Vibrio cholerae pathogenic cycle	0.048892513	1	43	K03087
*ko03020*	RNA polymerase	0.043861709	1	51	K03006
ko04115	p53 signaling pathway	0.036197553	1	59	K10808
*ko00620*	Pyruvate metabolism	0.034831078	1	75	K01572
ko04141	Protein processing in endoplasmic reticulum	0.027176857	1	140	K09503
ko03010	Ribosome	0.026398087	1	144	K02963
*ko02010*	ABC transporters	0.019687532	1	363	K02040
ko05168	Herpes simplex infection	0.017753549	1	126	K03006
*ko05016*	Huntington’s disease	0.015013068	1	149	K03006
ko05169	Epstein-Barr virus infection	0.014912981	1	150	K03006

Three pathways (metabolic pathways – ko01100; microbial metabolism in diverse environments – ko01120; and biosynthesis of secondary metabolites – ko01110) were excluded from this table, as they stand for higher functional hierarchy and overlap with many other KEGG metabolic pathways. Pathways italicize were found to be enriched in the Line Islands virome as well.

While we do expect contamination with cellular sequences in the viral-size fraction metagenomic set [32], we observed a significant overlap between the two enriched groups of pathways (39 pathways that were found enriched in the VirMic dataset were found enriched in the Line Islands virome, *P* value = 1.6 × 10^-20^, Additional file 2: Figure S1; overlapping pathways are italicized in Table 1). The Line Islands metagenomes consist of DNA reads having an average length of ~100 bp that yield less reliable KO annotations compared to the VirMic annotations (read lengths are 900–1000 bp). Therefore, considering the high overlap between the groups of enriched pathways, we decided to use only the VirMic annotations for further analysis.

### Global metabolism map and network

**Figure 2:**
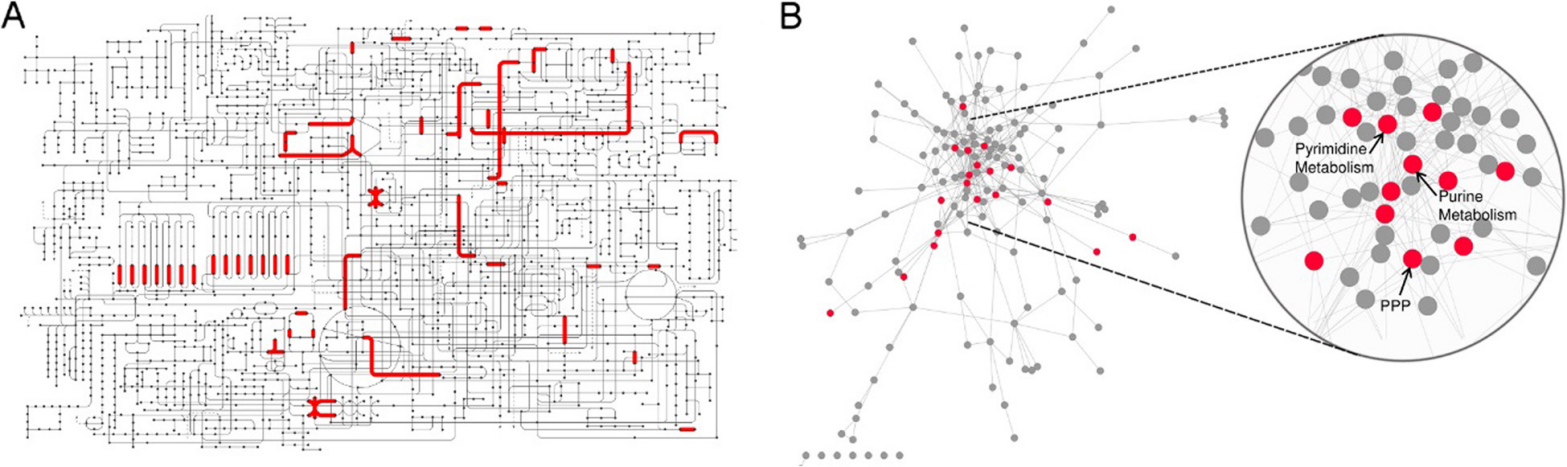
**Global metabolism map and network. (A)** KEGG Global Metabolism Map. Red edges represent KOs enriched in VirMic. Nodes represent metabolic compounds. **(B)** A network display of the Global Metabolism Map. Nodes represent specific KEGG metabolic pathways, edges represent metabolites shared by two pathways. Red nodes represent pathways enriched in VirMic. The enlarged part of the network encompasses the central pathways of the network.

One of the most comprehensive maps in the KEGG database is the global metabolism map, which combines the metabolic processes of 148 different metabolic pathways. Assigning the viral-enriched KOs to the global metabolism map showed local enrichment (i.e., only in specific areas of the map) (Figure 2A). Nevertheless, this map did not allow a systematic quantitative analysis of the metabolic functions found in the viral metagenome. To overcome the latter difficulty and allow an examination of the relationships between different viral-enriched pathways, we converted the Global Metabolism Map into a network in which each node represents a specific KEGG metabolic pathway and each edge denotes at least one metabolite used by two pathways (Figure 2B, see Methods). Moreover, moving to a graph representation enabled us to employ graph-based analyses that could not be conducted on the original global metabolic pathway map.

### Viral-enriched pathways play a central role in the global metabolism network

**Figure 3:**
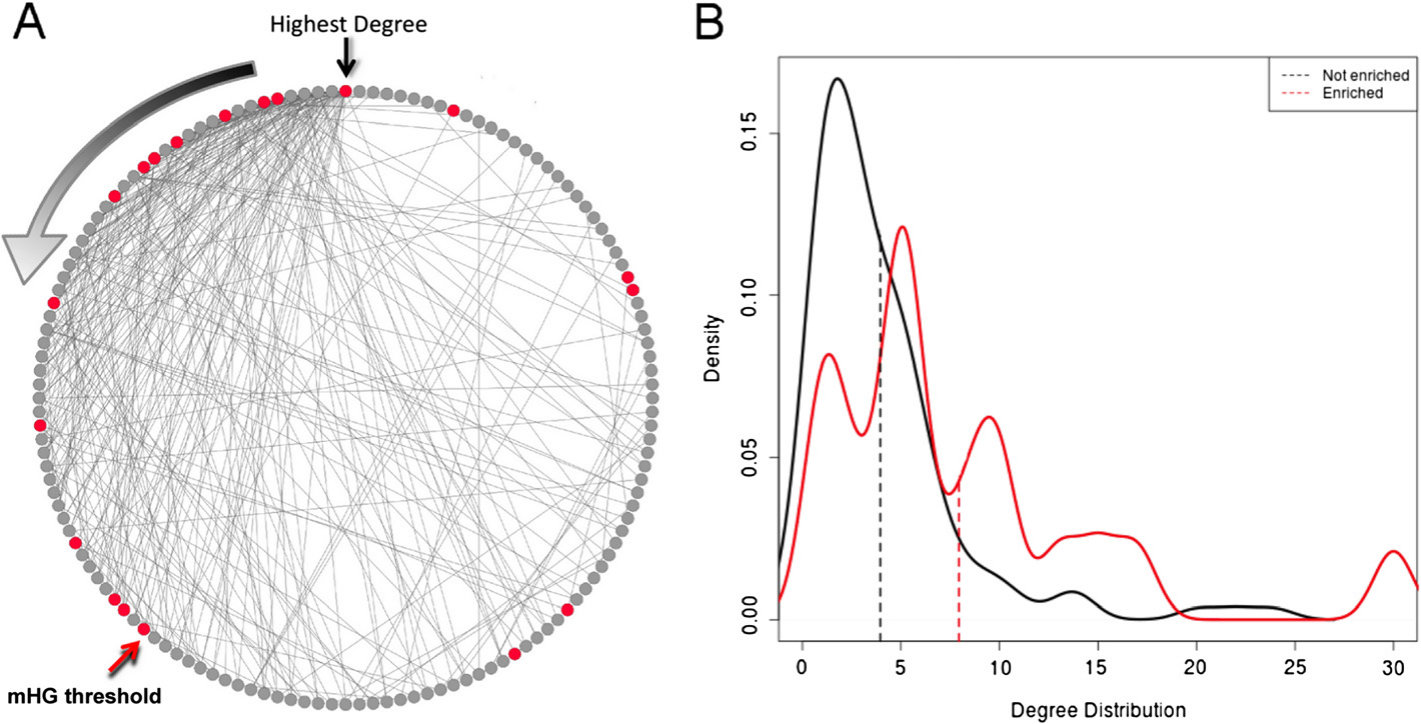
**VirMic enriched pathways show higher association with other metabolic pathways. (A)** A circle display of the pathways in the global metabolism network sorted according to network degree. Red nodes represent pathways enriched in VirMic. Black arrow indicates the pathway with the highest degree. Degree decreases in a counter-clockwise manner (gradient arrow). Red arrow represents the highest ranked pathways in which enrichment of viral pathways was detected. **(B)** Degree distribution of pathways enriched in viral reads (red) and pathways not enriched in viral reads (black). Dashed lines mark the means of the two distributions.

To unravel the role of viral-enriched pathways in the network, we sorted all pathways in the network according to degree, i.e., how many other pathways are connected to any given pathway (Figure 3A). Consequently, we used the minimal hypergeometric statistics [40] to evaluate the over-representation of viral-enriched pathways at the top of the ranked pathway list. We observed a significant over-representation (*P* = 6.3 × 10^-4^) of viral-enriched pathways among the 55 most connected nodes. When comparing the degree distributions of the viral-enriched and non-enriched pathways, we observed a significant difference in the two distributions, with a mean of 7.9 compared to 3.9 for the enriched and non-enriched pathways, respectively (*P* = 1.5 × 10^-3^, Mann–Whitney test). The higher degree of the viral-enriched pathways suggests that these pathways are central components of the global metabolism network and support key metabolic processes in any given cell.

### Pathways enriched in the viral metagenome are associated with purine and pyrimidine metabolism pathways

Two pathways having the highest enrichment scores were purine and pyrimidine metabolism pathways (Table 1). The importance of these pathways to viral replication has been demonstrated previously [26]. The presence of genes enhancing these functions was reported in both the genomes of cultured marine viruses [13,16] and in viromes [28]. We examined the association of purine and pyrimidine metabolism pathways with other pathways in the global metabolism network, especially other viral-enriched pathways. We hypothesized that a high association between purine and pyrimidine metabolism pathways and other enriched pathways might be the result of the enhancement of downstream metabolic processes that lead to purine and pyrimidine synthesis, while a low association can indicate that these pathways are independent.

**Figure 4:**
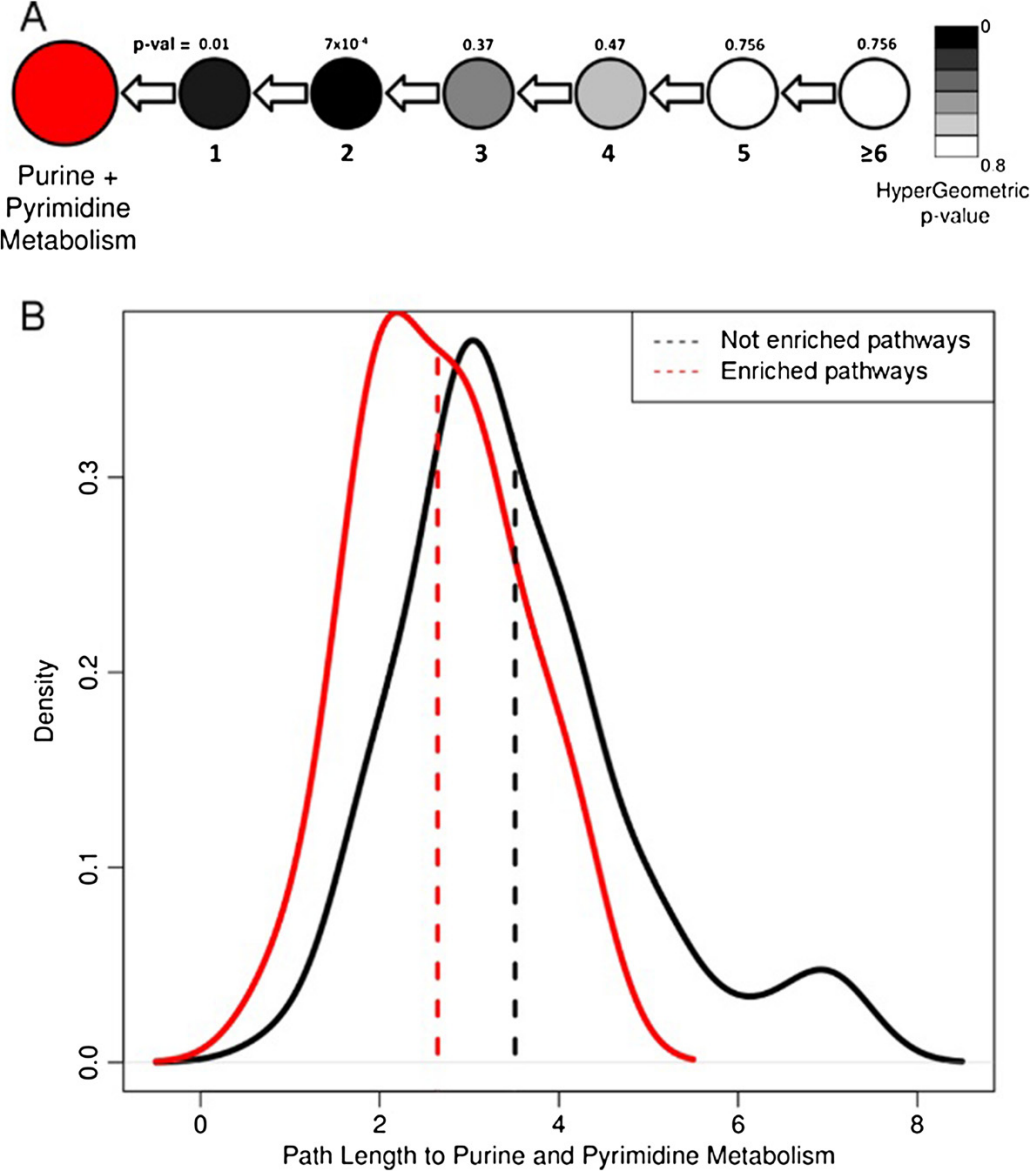
**Hypergeometric enrichment analysis of the number of steps connecting each metabolic pathway to purine and pyrimidine metabolism pathways. (A)** Red circle represents the purine and pyrimidine metabolism pathways. Lower numbers represent the path length to the purine and pyrimidine metabolism. Upper numbers represent the hypergeometric *P* value for viral enrichment in each path length. Black-white scale colors correspond to hypergeometric *P* values. **(B)** Path length distribution for the viral-enriched and non-enriched metabolic pathways.

To examine the relationship between the purine and pyrimidine metabolism pathways to other pathways, we measured the number of steps (path length) that connect each pathway in the global metabolism network to both purine and pyrimidine metabolism pathways. For example, pathways connected to purine and pyrimidine metabolism pathways via one step are the pathways that pass metabolites directly to purine and pyrimidine metabolism pathways, while pathways connected via two steps (path length = 2) pass metabolites to the pathways immediately upstream to purine and pyrimidine metabolism pathways, etc. We further counted the number of steps connecting each metabolic pathway to the purine and pyrimidine metabolism pathways and checked for over-representation of viral pathways in each step (Figure 4A). Overall, we observed a significant over-representation of viral-enriched pathways in the first and second steps, with hypergeometric *P* values of 0.01 and 7 × 10^-4^, respectively. A comparison of distributions of path lengths to purine and pyrimidine metabolism pathways for viral-enriched and non-viral pathways demonstrated that the viral-enriched pathways tend to be more closely associated with the purine and pyrimidine metabolism pathways (average number of steps was 2.6 compared to 3.5, *P* = 6 × 10^-4^, *t*-test) (Figure 4B).

Overall, as can be observed in the global metabolism network, most of the viral-enriched metabolic pathways are closely associated with purine and pyrimidine metabolism pathways. We hypothesize that the close association between enriched pathways and the relatively short path length connecting the enriched pathways to the purine and pyrimidine metabolism pathways results in a shift in cellular metabolic flux towards nucleotide synthesis. Therefore, it is possible that the fixation of metabolic pathways other than purine and pyrimidine metabolism also enhances nucleotide synthesis, and as a result, higher burst sizes and increased viral fitness.

While the existence of enzymes involved in purine and pyrimidine metabolism pathways in viral genomes and metagenomes has been reported previously [13,16,28], by employing a naïve pathway enrichment test, we were able to show quantitatively that these are the most significantly enriched pathways in viral metagenomes. The high enrichment score of these two pathways and the relatively high proportion of enriched KOs in the pathways (Table 1) reinforce that these two metabolic functions are highly important for viral replication and are fixed at the expense of increasing genome size.

As aforementioned, we found 13 viral-enriched KOs that belong to purine (Additional file 3: Figure S2) and pyrimidine (Additional file 4: Figure S3) metabolism pathways. An examination of the distribution of the enriched KOs within the purine and pyrimidine metabolism pathway graphs revealed that most of the reactions that are enhanced are located around the two end-products of these pathways, DNA and RNA. While this distribution can be explained by shifting the flux of these pathways towards the end-products, an appealing explanation is that the enhanced reactions promoting the incorporation of nucleic acids into DNA and RNA are bidirectional. Therefore, the enriched KOs might promote the degradation of the host genome and transcriptome in order to reuse its building blocks to replicate and transcribe the viral genome. This hypothesis is supported by the enrichment of enzymes promoting the conversion of nucleoside diphosphate (NDP) to deoxynucleoside diphosphate (dNDP) (ribonucleoside-diphosphate reductase alpha and beta chains (K00525, K00526), ribonucleoside-diphosphate reductase subunits M1 and M2 (K10807, K10808)). Genes enhancing the conversion of NDP to dNDP were observed previously in T4 phage [41] as well as in cyanophage genomes (S-PM2, P60), and it was assumed that they are potentially responsible for the scavenging of ribonucleotides for DNA synthesis [42,43].

### Pathways enriched in the viral metagenome

One of the viral-enriched metabolic pathways found to be directly associated with both purine and pyrimidine metabolism pathways (using the global metabolism network graph) is the pentose phosphate pathway. Genes belonging to the pentose phosphate pathway were detected previously in viruses infecting cultured cyanobacteria [13,23,24], including glucose-6-phosphate dehydrogenase (K00036), 6-phosphogluconate dehydorgenase (K00033), and transaldolase (K01623). Moreover, a recent study demonstrated that cyanophages carry a gene (*cp12*) that inhibits the Calvin cycle and therefore creates a shift in metabolic flux, and as a result enhances pentose phosphate pathway [23]. Two statistically-enriched KOs that are part of the pentose phosphate pathway were found enriched in this study: K01623 (transaldolase) and K01808 (RpiB). RpiB catalyzes the bidirectional conversion of D-ribulose 5-phosphate to D-ribose 5-phosphate; 6-phosphate dehydrogenase and 6-phosphogluconate dehydrogenase promote two of the three unidirectional reactions that result in elevated concentrations of D-ribulose 5-phosphate. Therefore, the substrate/products ratio for the reaction promoted by RpiB could result in the conversion of D-ribulose 5-phosphate to D-ribose 5-phosphate. Higher levels of D-ribose 5-phosphate can facilitate its conversion to 5-phosphoribosyl diphosphate, which is a compound used in purine and pyrimidine metabolism pathways, where it is converted to orothidine-5p by orotate phosphoribosyltransferase (*pyrE*, K00762), which was also enriched in the viral metagenome (Additional file 5: Figure S4). Therefore, we suggest that the viral-enriched enzymes of the pentose phosphate pathway promote nucleotide synthesis not only by increasing the generation of reducing power (NADPH), but also by higher synthesis rates of metabolic precursors to be processed in the purine and pyrimidine metabolism pathways.

We examined in detail the VirMic scaffolds containing the *rpiB* gene (JCVI_SCAF_1096627104658/1096628255974/1096627055600), and determined that they originate from cyanophage genomes based on their gene content (blastn against database containing the abundant SAR11 [30] and SAR116 [31] phages, updated in December 2012) and genomic composition. The presence of the *rpiB* gene in scaffolds associated with phages infecting cyanobacteria is of great interest as there is no evidence for the existence of *rpiB* in cyanobacteria. Nevertheless, *rpiA*, whose product promotes the same reaction, is found frequently in cyanobacteria genomes. Two main differences arise when inspecting these two paralogs, which surprisingly do not share a sequence or structural similarity [44]: the *rpiB* gene is significantly shorter than the *rpiA* gene (e.g., 450 nt compared to 660 nt in *Escherichia coli*), and its product was shown to be involved in the metabolism of the rare sugar allose in addition to ribose [45]. Based on the enrichment of *rpiB* in the viral subset, we suggest that the fixation of the *rpiB* gene in cyanophage genomes (and not *rpiA*) is preferred due to its shorter length, which allows replication, translation, and transcription at lower energy costs. This observation is consistent with previous studies, which demonstrated that viral gene length is about 2/3 of their bacterial homologs [23,46].

Among the enriched KOs in the viral metagenome, we also detected two enriched KOs that belong to two-component system pathways: K02040, the *pstS* gene related to phosphate sensing detected previously in marine cyanophage genomes [16]; and marine metagenomes [47] and K08350, the *fdnI* gene related to nitrate sensing [48]. Two-component systems are fundamental prokaryotic stimulus–response mechanisms that allow modifications in cellular gene expression and metabolism as a response to environmental conditions [49]. Cyanophage *pstS* was recently shown to be up-regulated in phosphate starvation conditions [50], and it was therefore suggested that fixation of this gene in phage genomes allows higher viral fitness through modification of the host P-acquisition. The formate-dehydrogenase-N gene (*fdnI*, K08350) is a member of the two-component system pathway that is regulated apparently by extra cellular nitrite/nitrate sensing [48]. We postulate that this member of the two-component system allows viral modification of the host metabolism in a similar manner to the *pstS* gene. While *pstS* genes are detected both in cultured and environmental marine cyanophage genomes, to the best of our knowledge, this is the first detection of viral enrichment of *fdnI* genes.

### KOs enriched in the viral metagenome promote sequential reactions within pathways

It has been hypothesized previously that auxiliary genes overcome metabolic bottlenecks within specific metabolic pathways [11]. To test this hypothesis, we examined in detail metabolic pathways that included the highest number of enriched KOs. Among them were four metabolic pathways that contained ≥4 enriched KOs: purine metabolism (KO00230); pyrimidine metabolism (KO00240); fructose and mannose metabolism (KO00051); and the amino sugar and nucleotide sugar metabolism (KO00520). Interestingly, we found that the enriched KOs tend to be clustered in specific regions of the metabolic pathways and often promote sequential reactions. Sequential reactions comprised of at least four reactions in which at most one KO is not enriched in the viral metagenome were defined as “metabolic motifs.” We calculated the probability of randomly finding such motifs within the enriched pathways assuming a binomial distribution. Employing successive *Bernoulli* experiments (see Methods), we identified 12 occurrences of significant motifs (Additional file 6: Table S2). The presence of viral-enriched metabolic motifs suggests that viral host-like genes do not only enhance reactions that are “metabolic bottlenecks” within pathways, but also promote metabolic processes with a higher degree of complexity.

Analysis of significant metabolic motifs in the different pathways revealed an interesting overlap between them. For example, the metabolic motif found in the fructose and mannose metabolism pathway (Additional file 7: Figure S5) is composed of the same KOs (in the same order) as the metabolic motif found in the amino sugar and nucleotide sugar metabolism pathway (Additional file 8: Figure S6). Such modularity was also found in the purine metabolism pathways (Additional file 3: Figure S2), where two metabolic motifs comprised of the same KOs were identified in the same order. We hypothesize that viral host-like genes are under the selection of two complementary forces: the first selective force favors the fixation of functional genes with a reduced gene length in order to minimize energy costs, as was previously suggested [23,46]; the second selective force favors the fixation of genes that promote critical reactions coordinately in a number of pathways, therefore allowing for the enhancement of two (or more) metabolic processes at the expense of fixing a single gene.

## Conclusions

Modifications of host metabolism by viruses, which result in elevated nucleotide biosynthesis, were suggested previously based on several cultured model viruses [26,51,52]. Our study, based on viral metagenomes representing the marine viral gene pool, demonstrates the importance of this metabolic function to viral fitness, as reflected by the high enrichment of the purine and pyrimidine metabolism pathways across the marine viral gene pool. Moreover, our analysis, based on the global metabolism network, suggests that many other viral-enriched pathways are closely associated with the purine and pyrimidine metabolism pathways. We also observed the enrichment of viral genes responsible for the degradation of cellular DNA and RNA, as well as for the conversion of ribonucleotides to deoxyribonucleotides. Therefore, we hypothesize that metabolic genes carried in marine viral genomes expand the nucleotide pool in infected hosts using two combined strategies: i) recycling the building blocks of the cellular genome and transcriptome; and ii) shifting the host metabolism in order to provide substrates for *de novo* synthesis of purine and pyrimidine.

## Abbreviations

dNDP: Deoxynucleoside diphosphate; GOS: Global Ocean Survey; KO: KEGG Ortholog; NDP: Nucleoside diphosphate.

## Competing interests

The authors declare that they have no competing interests.

## Authors’ contributions

HE, YM-G and OB designed the experiments. HE performed the bioinformatics and statistics. HE, YM-G and OB wrote the manuscript. All authors read and gave approval to the final manuscript.

## Supplementary Material

Additional file 1: Table S1 List of viral-enriched KEGG orthologs. Click here for file

Additional file 2: Figure S1 Venn diagram showing the overlap between the enriched pathways in VirMic and Line Islands virome. Out of 53 enriched pathways in VirMic, 39 were found to be enriched in the Line Islands virome, *P* = 1.6e-20. Click here for file

Additional file 3: Figure S2 Metabolic map for purine metabolism. Enzymes are denoted by their E.C. numbers, red squares represent viral-enriched Kos. Click here for file

Additional file 4: Figure S3 Metabolic map for pyrimidine metabolism. Enzymes are denoted by their E.C. numbers, red squares represent viral-enriched KOs. Click here for file

Additional file 5: Figure S4 Metabolic map for the pentose phosphate pathway. Enzymes are denoted by their E.C. numbers, red squares represent viral-enriched KOs. Click here for file

Additional file 6: Table S2 List of metabolic motifs found in viral-enriched pathways. Each motif is denoted by the KO number of the pathway it is related to. The *P* refers to the result of the statistical approach used to examine the significance for the appearance of each motif. Click here for file

Additional file 7: Figure S5 Metabolic map for fructose and manose metabolism. Enzymes are denoted by their E.C. numbers, red squares represent viral-enriched KOs. Click here for file

Additional file 8: Figure S6 Metabolic map for the amino sugar and nucleotide sugar metabolism pathway. Enzymes are denoted by their E.C. numbers, red squares represent viral-enriched KOs, pink squares represent KOs present in the viral metagenome that were not found to be enriched. Click here for file
