# Supplementary material for: Comparative metagenomic analyses reveal viral-induced shifts of host metabolism towards nucleotide biosynthesis
Source: Microbiome. 2014 Mar 26;2:9. doi: 10.1186/2049-2618-2-9 (PMC4022391; doi:10.1186/2049-2618-2-9)
Supplement: Additional file 1: Table S1 — List of viral-enriched KEGG orthologs. [file 2049-2618-2-9-S1.docx]

**Table S1**. List of viral enriched KEGG orthologs.

| **KEGG Ortholog** | **Name** | **Definition** | **GOS Scaffolds** | **Hyper Geometric P-value** | **number of hits in VirMic** |
| --- | --- | --- | --- | --- | --- |
| ***K00606*** | panB | 3-methyl-2-oxobutanoate hydroxymethyltransferase [EC:2.1.2.11] | JCVI_SCAF_1096627731713, JCVI_SCAF_1096627385149, JCVI_SCAF_1096626627396, JCVI_SCAF_1096626893752, JCVI_SCAF_1096627284915, JCVI_SCAF_1096627177339, JCVI_SCAF_1096627177529, | 1.00E-016 | 17 |
| ***K01185*** | E3.2.1.17 | lysozyme [EC:3.2.1.17] | JCVI_SCAF_1096627364882, JCVI_SCAF_1096627018658, JCVI_SCAF_1096627389241, | 1.00E-016 | 15 |
| ***K02371*** | fabK | enoyl-[acyl carrier protein] reductase II [EC:1.3.1.-] | JCVI_SCAF_1096626979423, JCVI_SCAF_1101668735076, JCVI_SCAF_1096626968704, JCVI_SCAF_1096627751594, JCVI_SCAF_1096627077249, JCVI_SCAF_1096627162261, | 1.00E-016 | 14 |
| ***K03111*** | ssb | single-strand DNA-binding protein | JCVI_SCAF_1096627510176, JCVI_SCAF_1096626627396, JCVI_SCAF_1096626923853, JCVI_SCAF_1101668183919, JCVI_SCAF_1096627231399, JCVI_SCAF_1096626632408, JCVI_SCAF_1096627522253, JCVI_SCAF_1096627502316, JCVI_SCAF_1096628036061, JCVI_SCAF_1096627823561, JCVI_SCAF_1096627385149, JCVI_SCAF_1096628385900, JCVI_SCAF_1096628201501, JCVI_SCAF_1096626659356, JCVI_SCAF_1096626893752, JCVI_SCAF_1096627177339, JCVI_SCAF_1096627504991, JCVI_SCAF_1096627695455, JCVI_SCAF_1096628111213, | 1.00E-016 | 27 |
| ***K03164*** | TOP2 | DNA topoisomerase II [EC:5.99.1.3] | JCVI_SCAF_1096626853028, JCVI_SCAF_1096627349372, JCVI_SCAF_1096626870105, | 1.00E-016 | 25 |
| ***K03257*** | EIF4A | translation initiation factor 4A | JCVI_SCAF_1096627348832, | 1.00E-016 | 11 |
| ***K03465*** | E2.1.1.148, | thymidylate synthase (FAD) [EC:2.1.1.148] | JCVI_SCAF_1096626864678, JCVI_SCAF_1096628310423, JCVI_SCAF_1096627531280, JCVI_SCAF_1096627080753, JCVI_SCAF_1096628167713, JCVI_SCAF_1096626862564, JCVI_SCAF_1096626911780, | 1.00E-016 | 15 |
| ***K07467*** | rstA1 | phage replication initiation protein | JCVI_SCAF_1096627389014, | 1.00E-016 | 8 |
| ***K10807*** | RRM1 | ribonucleoside-diphosphate reductase subunit M1 [EC:1.17.4.1] | JCVI_SCAF_1096626854395, | 1.00E-016 | 15 |
| ***K10755*** | RFC2_4 | replication factor C subunit 2/4 | JCVI_SCAF_1096627138013, JCVI_SCAF_1096627080753, JCVI_SCAF_1096626911780, JCVI_SCAF_1096626862564, | 5.00E-015 | 7 |
| ***K08350*** | fdnI | formate dehydrogenase-N, gamma subunit | JCVI_SCAF_1096627389014, | 1.93E-014 | 5 |
| ***K01768*** | E4.6.1.1 | adenylate cyclase [EC:4.6.1.1] | JCVI_SCAF_1096626907437, JCVI_SCAF_1096627289696, JCVI_SCAF_1096627089045, JCVI_SCAF_1096626915608, | 1.43E-013 | 10 |
| ***K02380*** | fdhE | FdhE protein | JCVI_SCAF_1096627389014, | 2.17E-013 | 6 |
| ***K00525*** | E1.17.4.1A, | ribonucleoside-diphosphate reductase alpha chain [EC:1.17.4.1] | JCVI_SCAF_1096626854395, JCVI_SCAF_1096626867376, JCVI_SCAF_1096627384817, | 7.06E-013 | 18 |
| ***K01623*** | ALDO, | fructose-bisphosphate aldolase, class I [EC:4.1.2.13] | JCVI_SCAF_1101668291686, JCVI_SCAF_1096627383283, JCVI_SCAF_1096627168531, JCVI_SCAF_1096627323705, JCVI_SCAF_1096626987692, JCVI_SCAF_1096627758744, JCVI_SCAF_1096627057106, JCVI_SCAF_1096627047369, JCVI_SCAF_1096627705816, JCVI_SCAF_1096627180231, JCVI_SCAF_1096626994794, | 7.83E-013 | 13 |
| ***K04801*** | rfcS | replication factor C small subunit | JCVI_SCAF_1096627284174, JCVI_SCAF_1096626907808, JCVI_SCAF_1096627166494, JCVI_SCAF_1096626852521, | 1.19E-012 | 7 |
| ***K04488*** | iscU, | nitrogen fixation protein NifU and related proteins | JCVI_SCAF_1096627839980, JCVI_SCAF_1096626854311, JCVI_SCAF_1096626853996, JCVI_SCAF_1096627160741, JCVI_SCAF_1101668279682, JCVI_SCAF_1096626945599, | 1.56E-012 | 9 |
| ***K01711*** | E4.2.1.47, | GDPmannose 4, 6-dehydratase [EC:4.2.1.47] | JCVI_SCAF_1096627282967, JCVI_SCAF_1096627296442, JCVI_SCAF_1096627313587, JCVI_SCAF_1096627679763, JCVI_SCAF_1096627556784, JCVI_SCAF_1096627014936, JCVI_SCAF_1096626848677, JCVI_SCAF_1096628390190, | 5.45E-012 | 13 |
| ***K05575*** | ndhD | NAD(P)H-quinone oxidoreductase subunit 4 [EC:1.6.5.3] | JCVI_SCAF_1096627181117, JCVI_SCAF_1096627385003, JCVI_SCAF_1096627179179, JCVI_SCAF_1096627156275, | 8.79E-012 | 9 |
| ***K01854*** | glf | UDP-galactopyranose mutase [EC:5.4.99.9] | JCVI_SCAF_1096627023014, JCVI_SCAF_1096626907808, | 4.91E-011 | 6 |
| ***K03217*** | yidC, | preprotein translocase subunit YidC | JCVI_SCAF_1096627356937, JCVI_SCAF_1096626888333, | 1.02E-010 | 10 |
| ***K02703*** | psbA | photosystem II P680 reaction center D1 protein | JCVI_SCAF_1096627012170, JCVI_SCAF_1096627965504, JCVI_SCAF_1096626956250, JCVI_SCAF_1101668699797, JCVI_SCAF_1096627572272, JCVI_SCAF_1096627016144, JCVI_SCAF_1096627180827, | 1.12E-010 | 7 |
| ***K01079*** | serB, | phosphoserine phosphatase [EC:3.1.3.3] | JCVI_SCAF_1096627315158, | 1.66E-010 | 7 |
| ***K13281*** | uvsE, | UV DNA damage endonuclease [EC:3.-.-.-] | JCVI_SCAF_1096626854834, JCVI_SCAF_1096626959094, | 4.61E-010 | 6 |
| ***K03386*** | E1.11.1.15, | peroxiredoxin (alkyl hydroperoxide reductase subunit C) [EC:1.11.1.15] | JCVI_SCAF_1096627486536, JCVI_SCAF_1096626852842, JCVI_SCAF_1096627032801, JCVI_SCAF_1096628032750, JCVI_SCAF_1096627603677, JCVI_SCAF_1096627398054, | 1.29E-009 | 7 |
| ***K12046*** | TNNT3 | troponin T, fast skeletal muscle | JCVI_SCAF_1096626854834, | 1.43E-009 | 3 |
| ***K06445*** | fadE | acyl-CoA dehydrogenase [EC:1.3.99.-] | JCVI_SCAF_1096626854116, | 1.14E-008 | 5 |
| ***K02040*** | pstS | phosphate transport system substrate-binding protein | JCVI_SCAF_1096626953943, JCVI_SCAF_1096627513414, JCVI_SCAF_1096627381361, JCVI_SCAF_1096626938230, JCVI_SCAF_1096628385461, JCVI_SCAF_1096626985784, JCVI_SCAF_1096626970597, | 7.14E-008 | 7 |
| ***K02706*** | psbD | photosystem II P680 reaction center D2 protein | JCVI_SCAF_1096627317553, JCVI_SCAF_1096627047510, JCVI_SCAF_1096627145129, JCVI_SCAF_1096626987982, | 1.94E-007 | 5 |
| ***K12418*** | K12418 | fatty acid desaturase (delta-4 desaturase) [EC:1.14.19.-] | JCVI_SCAF_1096626854116, | 2.54E-007 | 2 |
| ***K02604*** | ORC2 | origin recognition complex subunit 2 | JCVI_SCAF_1096627255261, | 4.24E-007 | 2 |
| ***K01894*** | gluQ | glutamyl-Q tRNA(Asp) synthetase [EC:6.1.1.-] | JCVI_SCAF_1096627355747, | 8.92E-007 | 4 |
| ***K01809*** | E5.3.1.8, | mannose-6-phosphate isomerase [EC:5.3.1.8] | JCVI_SCAF_1096626907808, | 2.72E-006 | 6 |
| ***K00762*** | pyrE | orotate phosphoribosyltransferase [EC:2.4.2.10] | JCVI_SCAF_1096627055207, JCVI_SCAF_1096627375293, JCVI_SCAF_1096627055627, | 3.36E-006 | 6 |
| ***K00558*** | E2.1.1.37, | DNA (cytosine-5-)-methyltransferase [EC:2.1.1.37] | JCVI_SCAF_1096626902801, JCVI_SCAF_1096627684793, JCVI_SCAF_1096627146939, JCVI_SCAF_1096627282997, | 5.30E-006 | 6 |
| ***K00605*** | gcvT, | aminomethyltransferase [EC:2.1.2.10] | JCVI_SCAF_1096627388037, JCVI_SCAF_1101668308796, JCVI_SCAF_1096627055138, JCVI_SCAF_1096626989824, JCVI_SCAF_1096627050166, | 6.78E-006 | 8 |
| ***K02314*** | dnaB | replicative DNA helicase [EC:3.6.4.12] | JCVI_SCAF_1096627510176, JCVI_SCAF_1096628385900, JCVI_SCAF_1096628036061, JCVI_SCAF_1096626923853, JCVI_SCAF_1096627695455, | 8.95E-006 | 8 |
| ***K02323*** | DPB2 | DNA polymerase II small subunit [EC:2.7.7.7] | JCVI_SCAF_1096627175513, | 1.86E-005 | 3 |
| ***K00979*** | kdsB | 3-deoxy-manno-octulosonate cytidylyltransferase (CMP-KDO synthetase) [EC:2.7.7.38] | JCVI_SCAF_1096627098003, | 2.17E-005 | 5 |
| ***K02377*** | E1.1.1.271, | GDP-L-fucose synthase [EC:1.1.1.271] | JCVI_SCAF_1096627313587, | 2.32E-005 | 5 |
| ***K00459*** | E1.13.12.16 | nitronate monooxygenase [EC:1.13.12.16] | JCVI_SCAF_1096626874170, JCVI_SCAF_1096627328767, | 4.39E-005 | 4 |
| ***K05337*** | fer | ferredoxin | JCVI_SCAF_1096626656929, JCVI_SCAF_1096627375096, JCVI_SCAF_1096628000696, | 1.30E-004 | 3 |
| ***K09503*** | DNAJA2 | DnaJ homolog subfamily A member 2 | JCVI_SCAF_1096627348773, | 1.57E-004 | 2 |
| ***K02963*** | RP-S18, | small subunit ribosomal protein S18 | JCVI_SCAF_1096626659356, | 1.58E-004 | 4 |
| ***K01462*** | PDF, | peptide deformylase [EC:3.5.1.88] | JCVI_SCAF_1096626951009, JCVI_SCAF_1096627742493, JCVI_SCAF_1096627011531, JCVI_SCAF_1096626245080, JCVI_SCAF_1096627570656, | 2.90E-004 | 5 |
| ***K02334*** | dpo | DNA polymerase bacteriophage-type [EC:2.7.7.7] | JCVI_SCAF_1096627215520, JCVI_SCAF_1096628170308, | 3.03E-004 | 4 |
| ***K01808*** | rpiB | ribose 5-phosphate isomerase B [EC:5.3.1.6] | JCVI_SCAF_1096627104658, | 3.23E-004 | 3 |
| ***K03272*** | gmhC, | D-beta-D-heptose 7-phosphate kinase / D-beta-D-heptose 1-phosphate adenosyltransferase [EC:2.7.1.167 2.7.7.70] | JCVI_SCAF_1096626972106, | 3.34E-004 | 3 |
| ***K11179*** | tusE, | tRNA 2-thiouridine synthesizing protein E [EC:2.8.1.-] | JCVI_SCAF_1096627429578, | 3.77E-004 | 2 |
| ***K00472*** | E1.14.11.2 | prolyl 4-hydroxylase [EC:1.14.11.2] | JCVI_SCAF_1096627167647, | 3.88E-004 | 2 |
| ***K00939*** | E2.7.4.3, | adenylate kinase [EC:2.7.4.3] | JCVI_SCAF_1096626952198, JCVI_SCAF_1096626921742, | 4.10E-004 | 4 |
| ***K00612*** | E2.1.3.- | carbamoyltransferase [EC:2.1.3.-] | JCVI_SCAF_1096627156527, JCVI_SCAF_1096627009191, JCVI_SCAF_1096626962337, | 5.50E-004 | 4 |
| ***K00526*** | E1.17.4.1B, | ribonucleoside-diphosphate reductase beta chain [EC:1.17.4.1] | JCVI_SCAF_1101668510950, JCVI_SCAF_1096627032617, JCVI_SCAF_1096628376142, | 5.74E-004 | 4 |
| ***K01710*** | E4.2.1.46, | dTDP-glucose 4, 6-dehydratase [EC:4.2.1.46] | JCVI_SCAF_1096626961316, JCVI_SCAF_1096627158059, | 7.00E-004 | 5 |
| ***K00860*** | cysC | adenylylsulfate kinase [EC:2.7.1.25] | JCVI_SCAF_1096627138013, | 1.20E-003 | 3 |
| ***K06214*** | csgG | curli production assembly/transport component CsgG | JCVI_SCAF_1096626909592, | 2.35E-003 | 2 |
| ***K01572*** | E4.1.1.3B, | oxaloacetate decarboxylase, beta subunit [EC:4.1.1.3] | JCVI_SCAF_1096627251362, | 2.44E-003 | 2 |
| ***K01493*** | comEB | dCMP deaminase [EC:3.5.4.12] | JCVI_SCAF_1096627844348, JCVI_SCAF_1096626868761, | 3.11E-003 | 2 |
| ***K03642*** | rlpA | rare lipoprotein A | JCVI_SCAF_1096627196549, | 3.28E-003 | 2 |
| ***K02335*** | DPO1, | DNA polymerase I [EC:2.7.7.7] | JCVI_SCAF_1096627058519, JCVI_SCAF_1096627282997, | 3.28E-003 | 6 |
| ***K00124*** | E1.2.1.2B1 | formate dehydrogenase, beta subunit | JCVI_SCAF_1096627389014, | 3.38E-003 | 2 |
| ***K05580*** | ndhI | NAD(P)H-quinone oxidoreductase subunit I [EC:1.6.5.3] | JCVI_SCAF_1101668699781, JCVI_SCAF_1096627089417, | 3.61E-003 | 2 |
| ***K00971*** | manC, | mannose-1-phosphate guanylyltransferase [EC:2.7.7.13] | JCVI_SCAF_1096627268413, JCVI_SCAF_1096627071151, JCVI_SCAF_1096627409136, | 3.68E-003 | 3 |
| ***K13256*** | psiE | protein PsiE | JCVI_SCAF_1096627330481, | 3.74E-003 | 2 |
| ***K03006*** | RPB1, | DNA-directed RNA polymerase II subunit RPB1 [EC:2.7.7.6] | JCVI_SCAF_1096627563526, JCVI_SCAF_1096627643471, | 5.79E-003 | 2 |
| ***K10808*** | RRM2 | ribonucleoside-diphosphate reductase subunit M2 [EC:1.17.4.1] | JCVI_SCAF_1096626869772, JCVI_SCAF_1096628038475, | 7.32E-003 | 2 |
| ***K13628*** | iscA, | iron-sulfur cluster assembly protein | JCVI_SCAF_1096627041884, JCVI_SCAF_1096626934369, | 7.73E-003 | 2 |
| ***K03087*** | SIG2, | RNA polymerase nonessential primary-like sigma factor | JCVI_SCAF_1096627369111, | 7.90E-003 | 2 |
