# Supplementary figures and images for: Comparative metagenomic analyses reveal viral-induced shifts of host metabolism towards nucleotide biosynthesis
Source: Microbiome. 2014 Mar 26;2:9. doi: 10.1186/2049-2618-2-9 (PMC4022391; doi:10.1186/2049-2618-2-9)

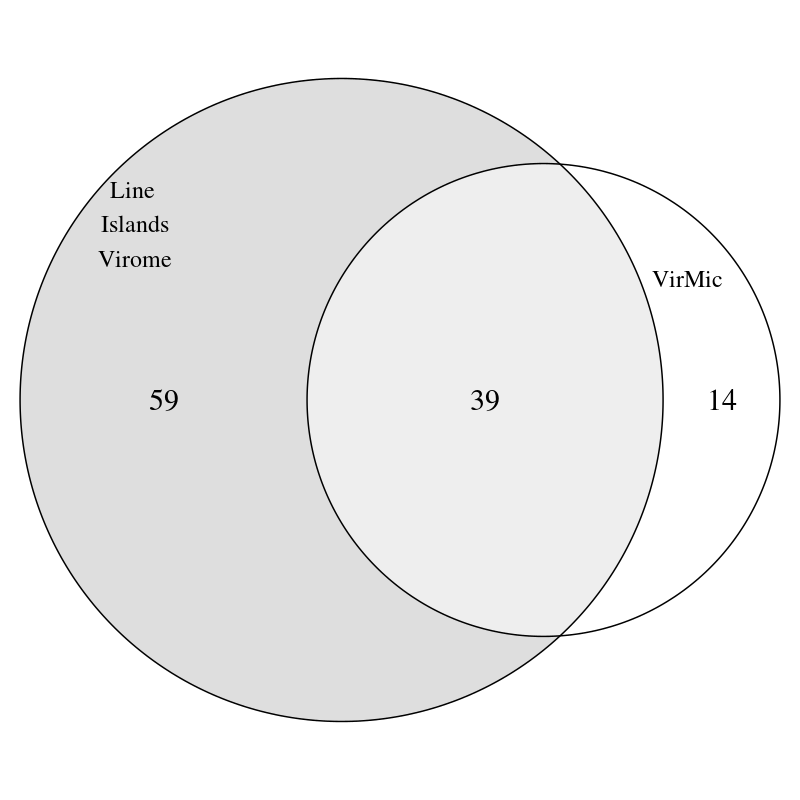

Supplement: Additional file 2: Figure S1 — Venn diagram showing the overlap between the enriched pathways in VirMic and Line Islands virome. Out of 53 enriched pathways in VirMic, 39 were found to be enriched in the Line Islands virome, P = 1.6e-20. [file 2049-2618-2-9-S2.tiff]

## PURINE METABOLISM

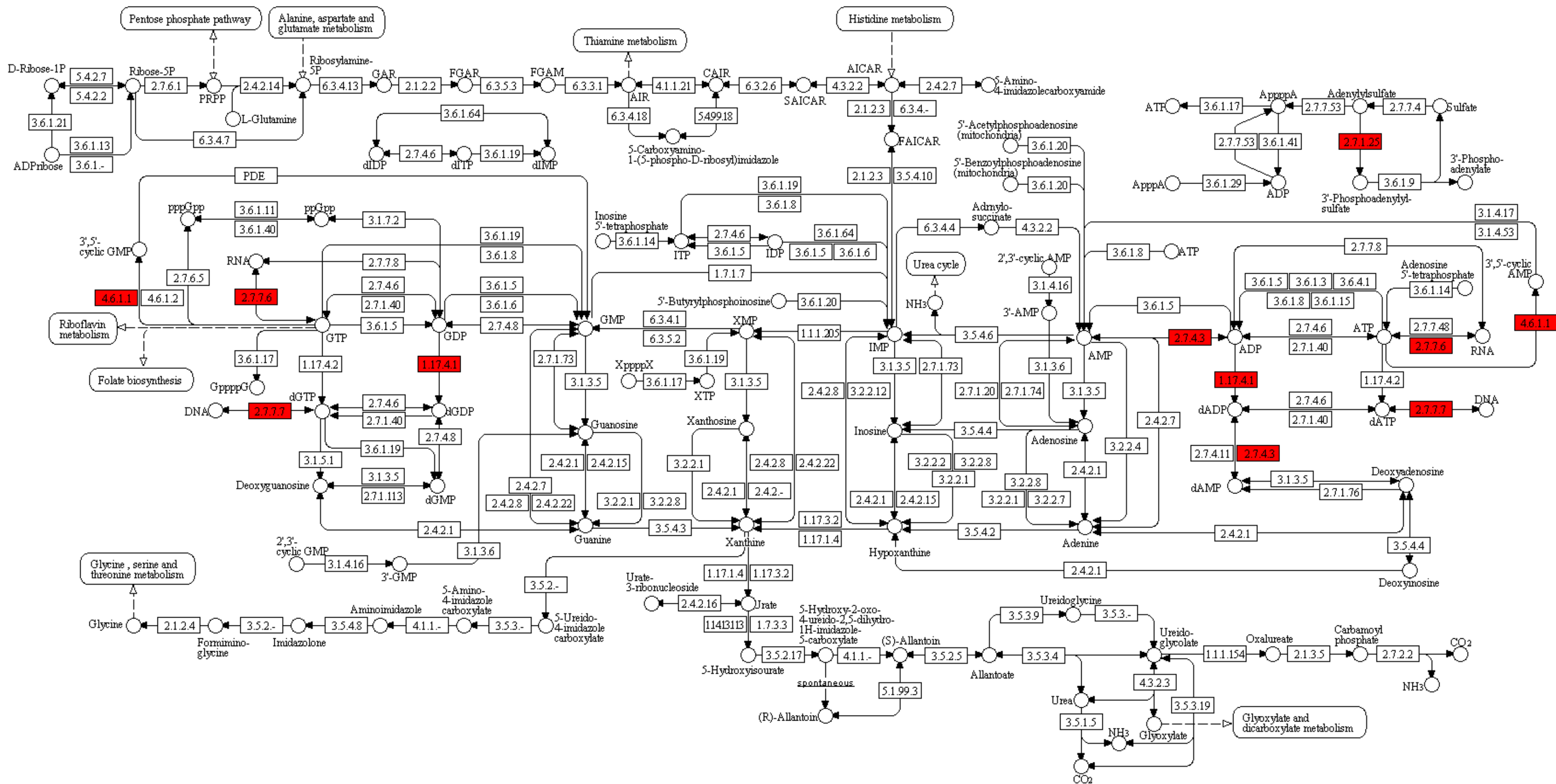

Supplement: Additional file 3: Figure S2 — Metabolic map for purine metabolism. Enzymes are denoted by their E.C. numbers, red squares represent viral-enriched Kos. [file 2049-2618-2-9-S3.pdf]

## PYRIMIDINE METABOLISM

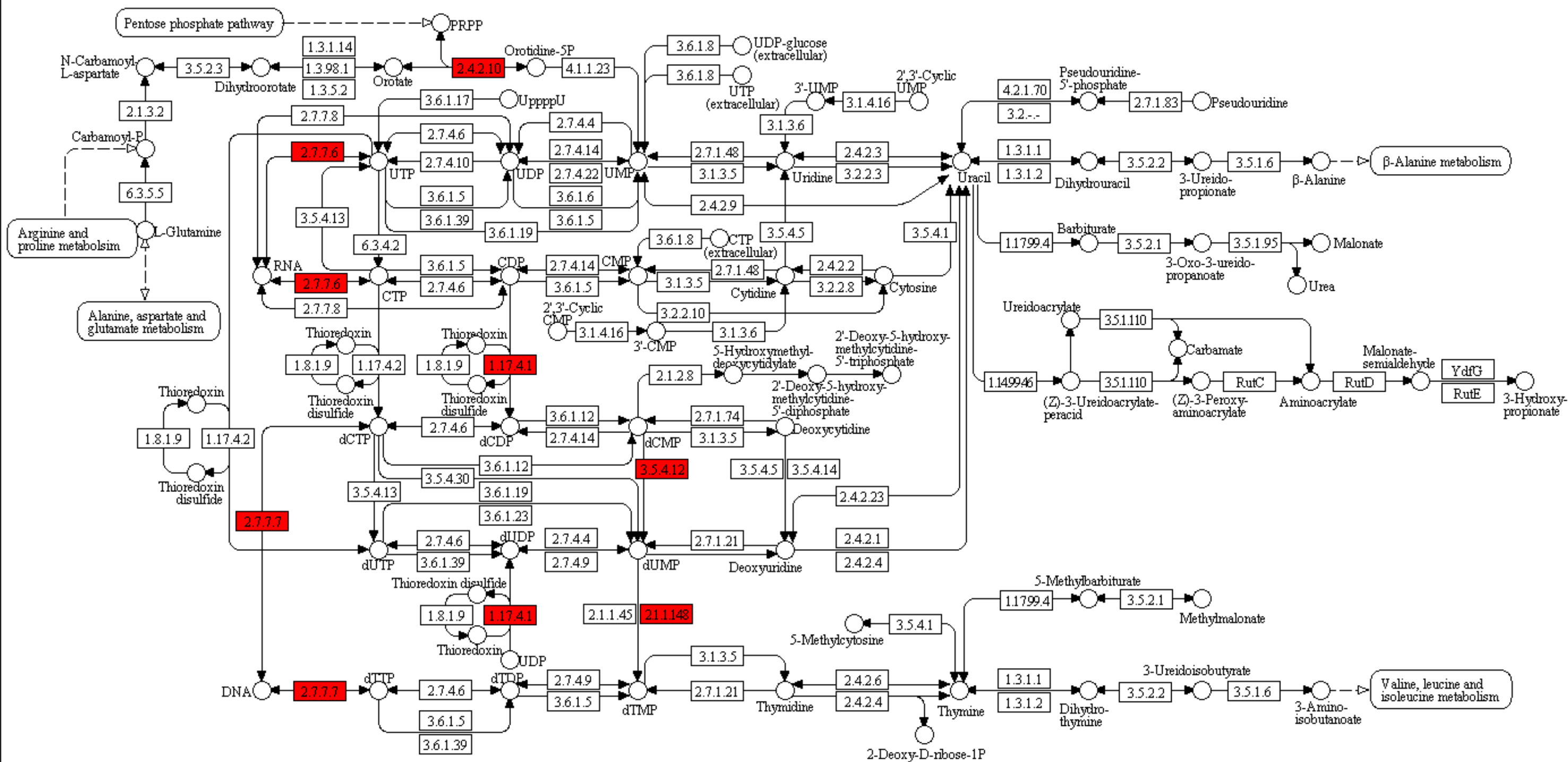

Supplement: Additional file 4: Figure S3 — Metabolic map for pyrimidine metabolism. Enzymes are denoted by their E.C. numbers, red squares represent viral-enriched KOs. [file 2049-2618-2-9-S4.pdf]

## PENTOSE PHOSPHATE PATHWAY

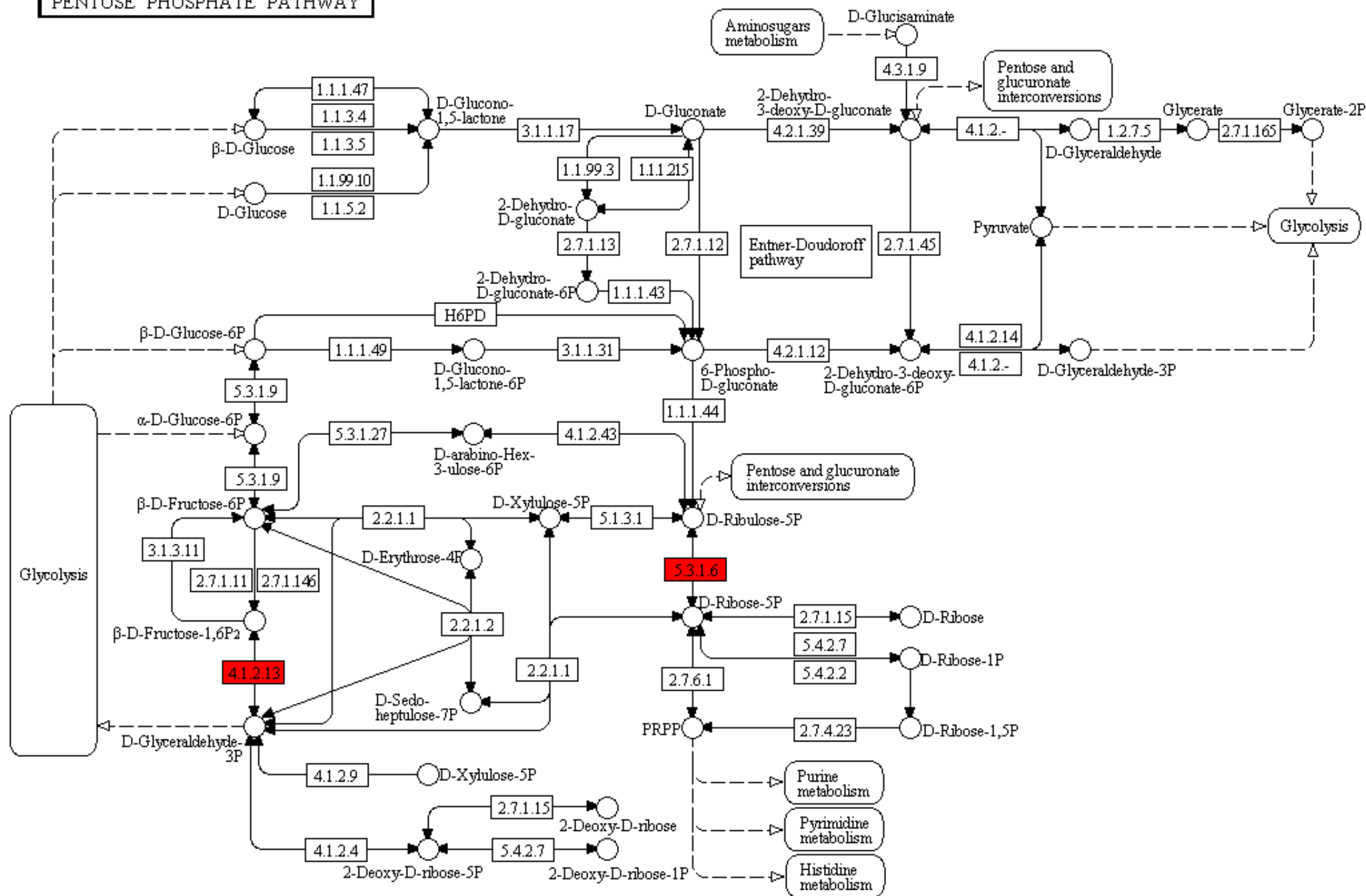

Supplement: Additional file 5: Figure S4 — Metabolic map for the pentose phosphate pathway. Enzymes are denoted by their E.C. numbers, red squares represent viral-enriched KOs. [file 2049-2618-2-9-S5.pdf]

# FRUCTOSE AND MANNOSE METABOLISM

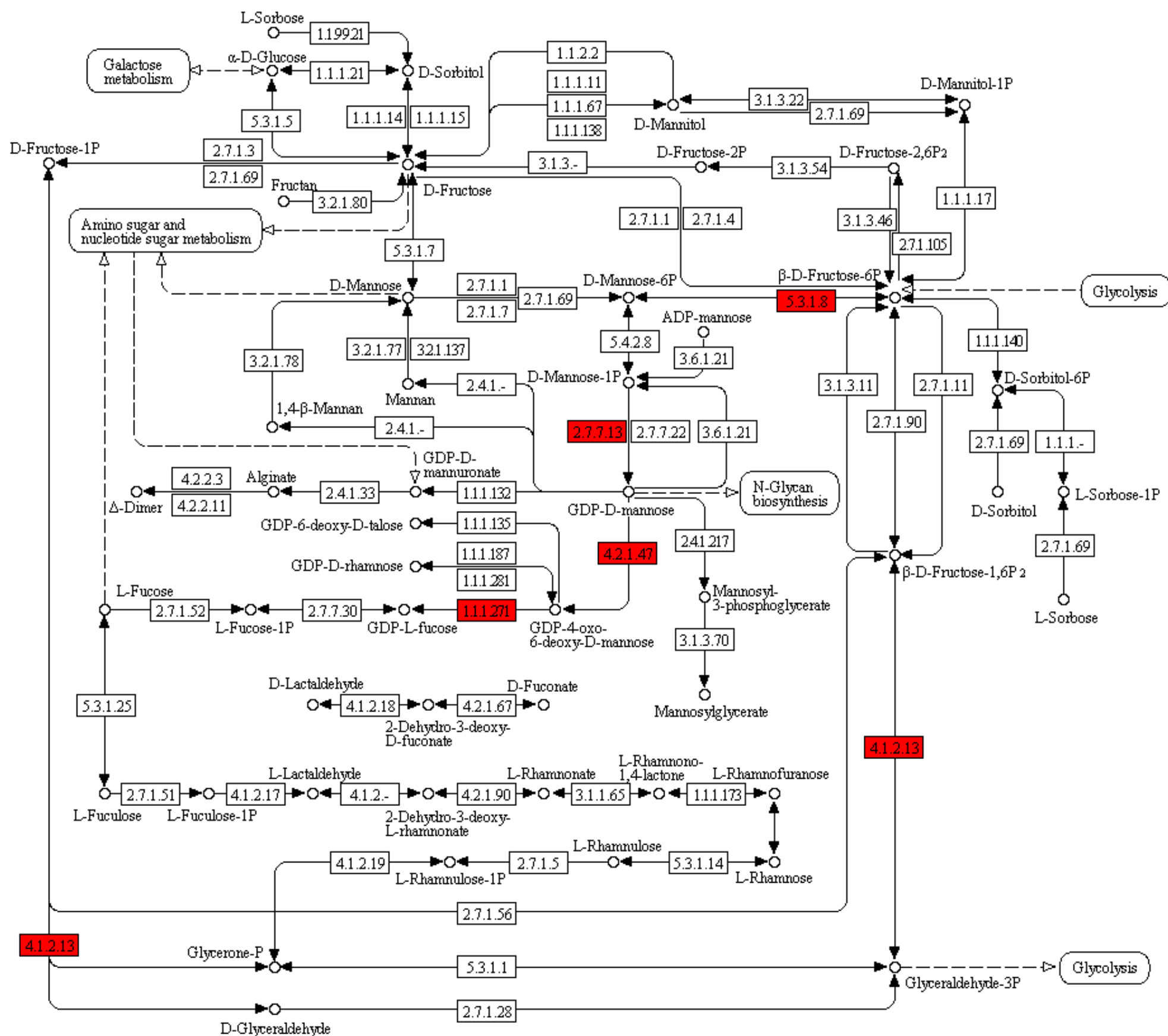

Supplement: Additional file 7: Figure S5 — Metabolic map for fructose and manose metabolism. Enzymes are denoted by their E.C. numbers, red squares represent viral-enriched KOs. [file 2049-2618-2-9-S7.pdf]
