# Supplementary material for: Comparative metagenomic analyses reveal viral-induced shifts of host metabolism towards nucleotide biosynthesis
Source: Microbiome. 2014 Mar 26;2:9. doi: 10.1186/2049-2618-2-9 (PMC4022391; doi:10.1186/2049-2618-2-9)
Supplement: Additional file 6: Table S2 — List of metabolic motifs found in viral-enriched pathways. Each motif is denoted by the KO number of the pathway it is related to. The P refers to the result of the statistical approach used to examine the significance for the appearance of each motif. [file 2049-2618-2-9-S6.docx]

**Table S2.** List of metabolic motifs found in viral enriched pathways. Each motif is denoted by the KO number of the pathway it is related to. The P-Value refers to the result of the statistical approach used to examine the significance for the appearance of each motif.

| **Pathway/motif** | **name** | **number of viral promoted reactions in the pathway** | **total reactions in the pathway** | **Ortholog 1** | **Ortholog 2** | **Ortholog 3** | **Ortholog 4** | **Ortholog 5** | **P-value** |
| --- | --- | --- | --- | --- | --- | --- | --- | --- | --- |
| KO00520 | amino sugar and nt suger metabolism | 4 | 95 | 1.1.1.271 | 4.2.1.47 | 2.7.7.13 | 5.4.2.8 | 5.3.1.8 | 1.02E-004 |
| KO00051 | fructose and manose | 6 | 51 | 1.1.1.271 | 4.2.1.47 | 2.7.7.13 | 5.4.2.8 | 5.3.1.8 | 1.97E-003 |
| KO00230-1 | purine metabolism | 11 | 100 | 2.7.7.6 | 4.6.1.1/3.6.1.5 | 1.17.4.1 |  |  | 1.43E-002 |
| KO00230-2 |  |  |  | 2.7.7.6 | 4.6.1.1/2.7.4.6 | 2.7.4.3/1.17.4.1 | 2.7.4.3 |  | 6.23E-004 |
| KO00230-3 |  |  |  | 2.7.7.7 | 1.17.4.2 | 2.7.7.6/4.6.1.1 |  |  | 8.62E-003 |
| KO00230-4 |  |  |  | 2.7.7.7 | 1.17.4.2 | 2.7.7.6/4.6.1.1 |  |  | 7.39E-003 |
| KO00230-5 |  |  |  | 2.7.4.3 | 1.17.4.1 | 2.7.4.3 |  |  | 4.18E-003 |
| KO00230-6 |  |  |  | 2.7.4.3 | 1.17.4.1 | 2.7.4.6 | 2.7.7.7 |  | 8.16E-004 |
| KO00240-1 | pyrimidine | 9 | 65 | 2.7.7.7 | 2.7.7.7 | 2.7.4.6 | 1.17.4.1 |  | 4.35E-003 |
| KO00240-2 |  |  |  | 2.7.7.6 | 2.7.7.6 | 2.7.4.6 | 1.17.4.1 |  | 6.21E-003 |
| KO00240-3 |  |  |  | 1.17.4.1 | 3.6.1.12 | 3.5.4.12 | 2.1.1.143 |  | 1.31E-002 |
| KO00240-4 |  |  |  | 3.5.4.12 | 21.1.1.143 | 2.7.4.9 | 1.17.4.1 |  | 9.12E-003 |
