# Supplementary material for: Comparative metagenomic analyses reveal viral-induced shifts of host metabolism towards nucleotide biosynthesis
Source: Microbiome. 2014 Mar 26;2:9. doi: 10.1186/2049-2618-2-9 (PMC4022391; doi:10.1186/2049-2618-2-9)
Supplement: Additional file 8: Figure S6 — Metabolic map for the amino sugar and nucleotide sugar metabolism pathway. Enzymes are denoted by their E.C. numbers, red squares represent viral-enriched KOs, pink squares represent KOs present in the viral metagenome that were not found to be enriched. [file 2049-2618-2-9-S8.pdf]

AMINO SUGAR AND NUCLEOTIDE SUGAR METABOLISM

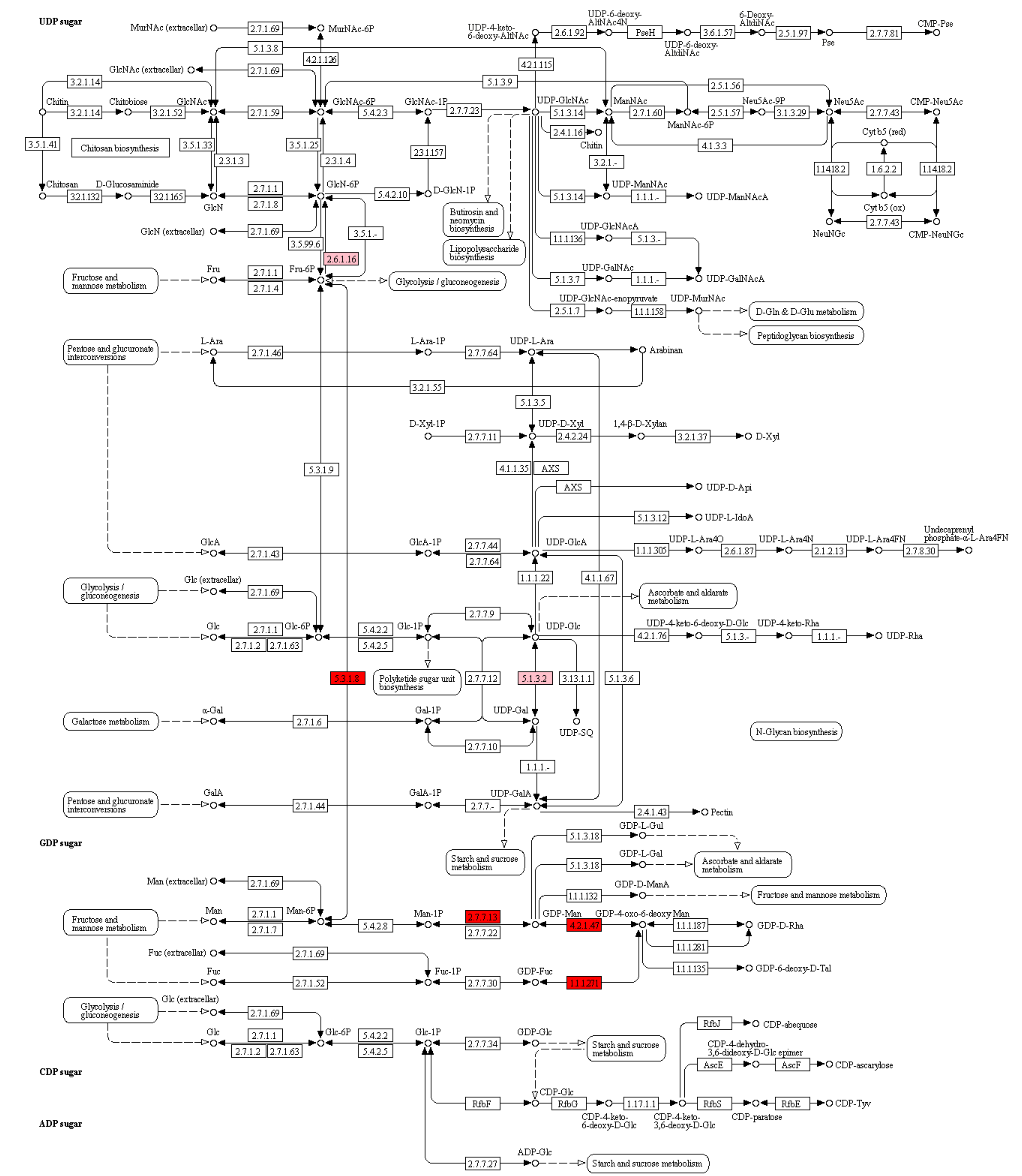

00520 7/30/12
(c) Kanehisa Laboratories
